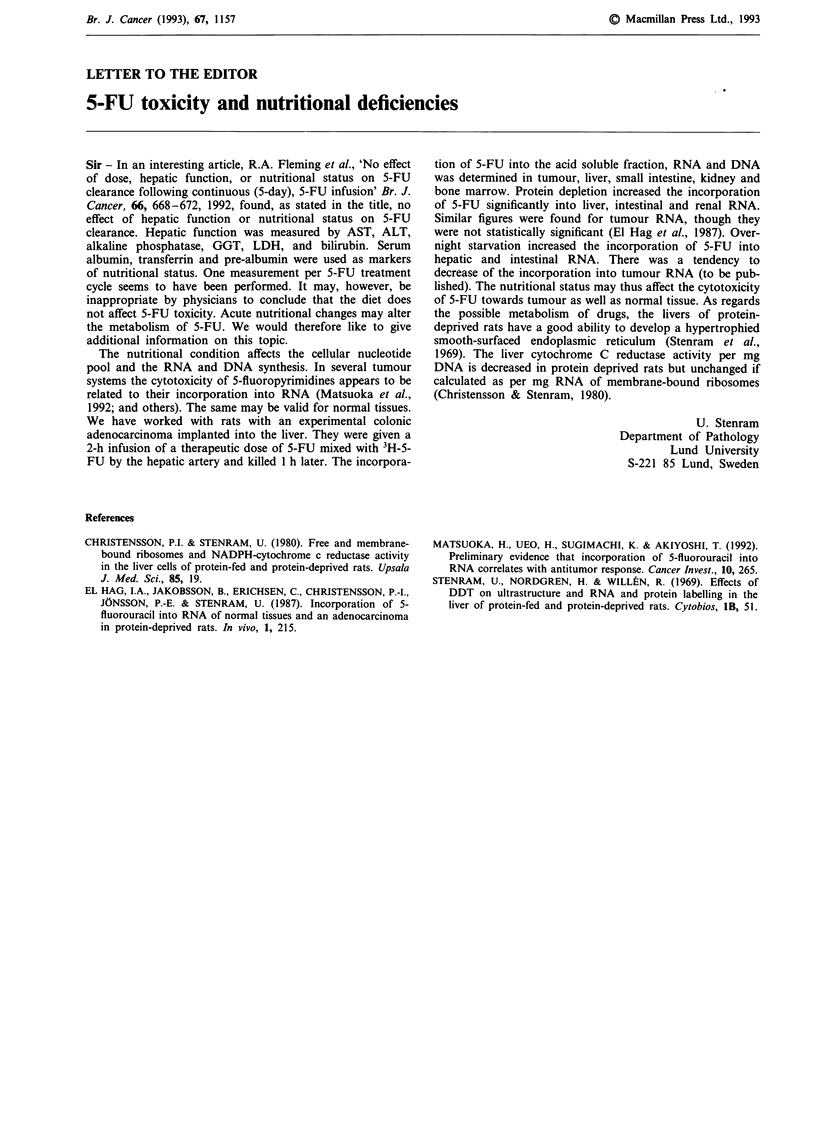# 5-FU toxicity and nutritional deficiencies.

**DOI:** 10.1038/bjc.1993.214

**Published:** 1993-05

**Authors:** U. Stenram


*Br. J. Cancer* (1993), **67**, 1157 © Macmillan Press Ltd., 1993

### LETTER TO THE EDITOR

### 5-FU toxicity and nutritional deficiencies

**Sir** – In an interesting article, R.A. Fleming *et al.*, 'No effect of dose, hepatic function, or nutritional status on 5-FU clearance following continuous (5-day), 5-FU infusion' *Br. J. Cancer,* **66,** 668–672, 1992, found, as stated in the title, no effect of hepatic function or nutritional status on 5-FU clearance. Hepatic function was measured by AST, ALT, alkaline phosphatase, GGT, LDH, and bilirubin. Serum albumin, transferrin and pre-albumin were used as markers of nutritional status. One measurement per 5-FU treatment cycle seems to have been performed. It may, however, be inappropriate by physicians to conclude that the diet does not affect 5-FU toxicity. Acute nutritional changes may alter the metabolism of 5-FU. We would therefore like to give additional information on this topic.

The nutritional condition affects the cellular nucleotide pool and the RNA and DNA synthesis. In several tumour systems the cytotoxicity of 5-fluoropyrimidines appears to be related to their incorporation into RNA (Matsuoka *et al.*, 1992; and others). The same may be valid for normal tissues. We have worked with rats with an experimental colonic adenocarcinoma implanted into the liver. They were given a 2-h infusion of a therapeutic dose of 5-FU mixed with $^{3}$H-5-FU by the hepatic artery and killed 1 h later. The incorporation of 5-FU into the acid soluble fraction, RNA and DNA was determined in tumour, liver, small intestine, kidney and bone marrow. Protein depletion increased the incorporation of 5-FU significantly into liver, intestinal and renal RNA. Similar figures were found for tumour RNA, though they were not statistically significant (El Hag *et al.*, 1987). Overnight starvation increased the incorporation of 5-FU into hepatic and intestinal RNA. There was a tendency to decrease of the incorporation into tumour RNA (to be published). The nutritional status may thus affect the cytotoxicity of 5-FU towards tumour as well as normal tissue. As regards the possible metabolism of drugs, the livers of protein-deprived rats have a good ability to develop a hypertrophied smooth-surfaced endoplasmic reticulum (Stenram *et al.*, 1969). The liver cytochrome C reductase activity per mg DNA is decreased in protein deprived rats but unchanged if calculated as per mg RNA of membrane-bound ribosomes (Christensson & Stenram, 1980).

U. Stenram
Department of Pathology
Lund University
S-221 85 Lund, Sweden

**References**

CHRISTENSSON, P.I. & STENRAM, U. (1980). Free and membrane-bound ribosomes and NADPH-cytochrome c reductase activity in the liver cells of protein-fed and protein-deprived rats. *Upsala J. Med. Sci.*, **85,** 19.

EL HAG, I.A., JAKOBSSON, B., ERICHSEN, C., CHRISTENSSON, P.-I., JÖNSSON, P.-E. & STENRAM, U. (1987). Incorporation of 5-fluorouracil into RNA of normal tissues and an adenocarcinoma in protein-deprived rats. *In vivo*, **1,** 215.

MATSUOKA, H., UEO, H., SUGIMACHI, K. & AKIYOSHI, T. (1992). Preliminary evidence that incorporation of 5-fluorouracil into RNA correlates with antitumor response. *Cancer Invest.*, **10,** 265.

STENRAM, U., NORDGREN, H. & WILLÉN, R. (1969). Effects of DDT on ultrastructure and RNA and protein labelling in the liver of protein-fed and protein-deprived rats. *Cytobios*, **1B,** 51.